# Schizophrenia susceptibility and NMDA-receptor mediated signalling: an association study involving 32 tagSNPs of *DAO*, *DAOA*, *PPP3CC*, and *DTNBP1* genes

**DOI:** 10.1186/1471-2350-14-33

**Published:** 2013-03-09

**Authors:** Emilio Sacchetti, Catia Scassellati, Alessandra Minelli, Paolo Valsecchi, Cristian Bonvicini, Patrizio Pasqualetti, Alessandro Galluzzo, Rosaria Pioli, Massimo Gennarelli

**Affiliations:** 1 Psychiatric Unit, University of Brescia, School of Medicine, Viale Europa 11, Brescia, 25123, Italy; 2 Department of Mental Health, Brescia Spedali Civili, Brescia, Italy; 3 Center of Behavioral and Neurodegenerative Disorders, Brescia University and EULO, Brescia, Italy; 4 Genetic Unit, I.R.C.C.S. “San Giovanni di Dio” – Fatebenefratelli, Via Pilastroni 4, Brescia, 25125, Italy; 5 Department of Molecular and Translational Medicine, Division of Biology and Genetics, Viale Europa 11, Brescia, 25123, Italy; 6 AFaR, Department of Neuroscience, Hospital Fatebenefratelli Isola Tiberina, Rome, 00186, Italy; 7 Psychitric Unit, I.R.C.C.S. “San Giovanni di Dio” - Fatebenefratelli, Brescia, Italy

**Keywords:** tagSNPs, Schizophrenia, Glutamate neurotransmission, DAO, DAOA, PPP3CC, DTNBP1, Association study, Phenotypic dissection

## Abstract

**Background:**

Recent studies supported associations between four NMDA-receptor-mediated signalling genes (D-amino acid oxidase, *DAO;* D-amino acid oxidase activator, *DAOA;* protein phosphatase 3 catalytic subunit gamma isoform, *PPP3CC;* dystrobrevin-binding protein 1, *DTNBP1*) and schizophrenia susceptibility, even though with contrasting results.

**Methods:**

In an attempt to replicate these findings for the first time in an Italian population, a panel of 32 tagSNPs was analysed in a representative case-control sample involving 879 subjects.

**Results:**

An association in the allele frequency was observed for the estimated *PPP3CC* CAG triplotype in the SNP window rs4872499 T/C-rs11780915 A/G-rs13271367 G/A (p_correct_ = 0.001). Similarly, the clustered genotype frequencies of the estimated/phased CAG triplotype differed between cases and controls (p = 0.004), with the carriers having a higher frequency in the control population (p = 0.002, odd ratio OR = 0.59, 95% confident interval CI: 0.43-0.82).

Following the phenotypic dissection strategy, the analysis of single SNPs evidenced a protective effect in males of rs11780915 and rs13271367 in *PPP3CC* gene (p_correct_ = 0.02, p_correct_ = 0.04 respectively). Moreover the estimated/phased GT diplotype (rs2070586A/G-rs3741775G/T) carriers of the *DAO* gene were more highly represented in female controls (p = 0.017, OR = 0.58, 95% CI: 0.37-0.90), as were the estimated/phased CAG triplotype carriers of the *PPP3CC* gene in females (p = 0.01, OR = 0.53*,* 95% CI: 0.32-0.87). In addition, we performed an interaction analysis, and a 66% (p = 0.003, OR = 0.34, 95% CI: 0.17-0.70) lower risk of developing schizophrenia for female (CAG + GT) carriers versus non-CAG or -GT carriers was observed. For *DTNBP1*, we found a protective effect in males for the rs6459409 (p_correct_ = 0.02) and the estimated/phased CT diplotype (rs6459409-rs9476886) carriers (p = 3x10^-4^*,* OR = 0.46*,* 95% CI: 0.30-0.70).

In relation to diagnostic subtypes, the estimated/phased *DAO* GT diplotype and *PPP3CC* CAG triplotype female carriers were found to show relative risk ratio (RRR) values of 0.52 and 0.54 lower risk for a paranoid phenotype respectively.

**Conclusions:**

Although the results are preliminary and needed replication in a larger sample, this study suggests that NMDA receptor-mediated signalling genes (*DAO, PPP3CC, DTNBP1*) might be involved in schizophrenia pathogenic mechanisms related to gender.

## Background

D-amino acid oxidase (*DAO*), D-amino acid oxidase activator (*DAOA*), protein phosphatase 3 catalytic subunit gamma isoform (*PPP3CC*), and dystrobrevin-binding protein 1 (*DTNBP1*) share the ability to modulate synaptic plasticity and glutamatergic transmission trough N-methyl-D-aspartate receptors (NMDARs) [1]. Because reduced synaptic plasticity and NMDAR hypofunction have been increasingly observed in the neurobiology of schizophrenia [2-4], the genes encoding *DAO*, *DAOA*, *PPP3CC*, and *DTNBP1* may be regarded as plausible candidates for schizophrenia susceptibility. Recent meta-analyses have supported this proposal, demonstrating an allelic association between the four genes and schizophrenia susceptibility [5-8], even though this evidence does not apply to all of the investigated single nucleotide polymorphisms (SNPs). A recent convergent functional genomics in schizophrenia has identified top genes among which *DTNBP1*[9]. In relation to the Italian population, no data are available with respect to these genes, except for a specific haplotype association of *DTNBP1* in a small sample report [10].

However, association studies have so far yielded equivocal results. Evidence that some SNPs of the *DAO*, *DAOA*, *PPP3CC*, and *DTNBP1* alleles contribute to conferring an increased risk of schizophrenia are counterbalanced by others indicating that they do not increase risk or even exert a protective effect (http://www.schizophreniaforum.org/res/sczgene). Moreover, the Psychiatric Genomics Consortium (PGC) recently published mega-analyses for schizophrenia including 9,394 cases and 12,462 controls that were combined in a single analysis, and the top 81 statistically independent loci from that analysis were then tested in over 8,000 cases [11]. This mega-analysis identified only seven significant loci that did not involve these four genes. A number of variables could have contributed to generate these inconsistent results. Genetic differences associated with ethnic background, as well as gender effects, the inherent phenotypic heterogeneity of schizophrenia, and the presence of epistatic or epigenetic interactions are rightly considered among the first-line putative sources of variation that should require a dedicated control; however, their influence on the putative associations between the *DAO*, *DAOA*, *PPP3CC*, and *DTNBP1* genes and schizophrenia unfortunately remains largely undetermined to date.

Motivated by these considerations, we planned a case-control association study that considered the possible confounding effects of ethnicity and gender, provided a reasonable phenotypic dissection of the patients and tested for the interactions. Our study evaluated 32 tagSNPs of the *DAO*, *DAOA*, *PPP3CC* and *DTNBP1* genes.

## Methods

### Case and control samples

The study involved a total of 879 subjects, which included 391 DSM-IV-TR - Diagnostic and Statistical Manual of Mental Disorders [12] schizophrenia patients and 488 healthy volunteers. Patients and controls were enrolled and diagnosed as described in detail elsewhere [13]. In particular patients and controls, enrolled in the study, gave written informed consent to the study participation according to the institutional guidelines of the local Ethic Committee (Fatebenefratelli hospital “San Giovanni di Dio” - Brescia, Italy), were of Caucasian heritage for at least two generations, lived in northern Italy, were unrelated to other prospective participants, and fulfilled predefined group-specific inclusion and exclusion criteria. A concise but unequivocal explanation about the aims of the study was included on the written consent form. The participants also received an explicit guarantee of anonymity. A unique number linked all the individual data.

The patients were enrolled from those voluntarily admitted to the Brescia University and Spedali Civili Psychiatric Unit or the Brescia IRCCS Fatebenefratelli. The inclusion criteria were a DSM-IV-TR diagnosis of schizophrenia and a level of understanding and attention judged sufficient to give true informed consent; a lifetime comorbidity with other DSM-IV-TR Axis I disorders was an exclusion criterion. All participants underwent detailed clinical interviews, implemented, when required, by DSM-IV-TR adjusted versions of the Structural Clinical Interview for DSM-IV Axis I Disorders, Clinician Version (SCID-CV) [14]. Patients were also evaluated about schizophrenia subtype and age at the onset of the disorder. To attribute the schizophrenia subtype, a checklist of the symptoms dominating the clinical picture at the screening visit and in the previous 4 weeks was used.

For the age at onset of schizophrenia, the appearance of the first psychotic symptoms represented the pre-identified operational cut-off; in order to reach as accurate estimates as possible, direct information from the patients was systematically retrieved along with data obtained from at least one close relative plus, when existing, previous medical records.

The controls were randomly recruited through different sources (hospital visitors, cultural and elderly associations, trade unions, word of mouth and newspaper advertising). They were screened for DSM-IV Axis I disorders by expert psychologists using the Mini-International Neuropsychiatric Interview (M.I.N.I.) [15]. Only healthy volunteers without a history of drug or alcohol abuse or dependence and without a personal or first-degree family history of psychiatric disorders were enrolled in the study. Subjects who obtained a score lower than 27/30 in the Mini Mental State Examination (M.M.S.E.) [16] were excluded as well.

When compared with controls, patients presented a younger age at the time of the genotyping (42.4 ± 12.5 vs. 45.4 ± 16.7 years, p < 0.01) and a higher proportion of males (67.8% vs. 43.9%, p < 0.001). The majority of the schizophrenia patients (60.1%) were affected by the paranoid subtype. Furthermore, among the 290 patients who provide sufficiently informative data, the age at the onset of schizophrenia was 23.8 ± 6.4 years, with an anticipatory effect for males with respect to females (23.2 ± 6.1 vs. 25.2 ± 6.8 years, p < 0.001).

Because controls and patients were not matched for sex or age, these variables were retained in the logistic/multinomial regression model analyses to control for potential confounding effects. The log-transformed age and age at onset were calculated to improving their approximation to a Gaussian distribution.

### Genotyping

The Puregene DNA purification kit (Gentra System) was used to extract DNA from each sample.

A panel of 47 SNPs were selected as tagging SNPs (by SNPbrowser version 3.5, [17]) from the HapMap Project (phase II, data release #21a, CEPH data set) to evenly encompass the regions of the *DAO*, *DAOA*, *PPP3CC*, and *DTNBP1* genes, including the 5’ and 3’ boundaries. The 47 SNPs panel was genotyped for the whole patient-control cohort using the SNPlex procedure in accordance with the current manufacturer’s protocol (Applied Biosystems, Foster City, CA, USA).

To minimise type I errors due to incorrect genotype assignment and excess of multiple tests, the inclusion of the SNPs in the association analyses was restricted to those with 1) no significant deviation from Hardy-Weinberg equilibrium (HWE) in both patients and controls (exact test p < 0.001); 2) a Minor Allele Frequency (MAF) greater than 0.1; 3) a completion rate for genotyping greater than 90%. 32 SNPs fulfilled these criteria after genotyping. A more in-depth description and a quality control were reported in Additional file 1.

Consequently, the analyses were conducted on samples of 862, 877, 861, and 855 individuals for *DAO* (N = 386 patients and N = 476 controls), *DAOA* (N = 391 patients and N = 486 controls), *PPP3CC* (N = 384 patients and N = 477 controls), and *DTNBP1* (N = 382 patients and N = 473 controls), respectively.

### Statistical analyses

PLINK software version 1.07 (http://pngu.mgh.harvard.edu/~purcell/plink) was used for HWE patient-control comparisons of all polymorphisms and phased haplotypes.

For the PLINK software, a nominal level of significance of p = 0.05 was accepted and corrected according to the 10000 permutation procedure for genotype frequencies and Bonferroni’s correction for allele frequencies for all genotype/allele association tests. In details these analyses were conducted separately for each gene considering 6 SNPs for DAO, 6 SNPs for DAOA, 8 SNPs for PPP3CC and 12 for DTNBP1.

Haploview program version 4.2 [18] was used to evaluate differences in allele frequencies, calculate the pairwise Linkage Disequilibrium (LD) between SNPs, and estimate haplotype frequencies for each block of linkage. Patient-control differences in haplotype distribution were determined by 10000 permutation test.

Case-control sliding window haplotypes, *χ*^2^ tests and 10000 permutation tests were performed utilising the Haploview program version 4.2. These window sizes were chosen to approximately examine haplotypes within the specific LD blocks identified by LD analyses. Moreover, the sliding window approach was chosen to systematically explore other haplotypes in areas of lower LD [18]. The corrections were performed considering each gene separately.

Only the allele frequencies of the haplotypes >5% were included in the analyses, those <5% the haplotypes were excluded.

Phased haplotypes aimed at assigning the two most plausible haplotypes to each sample were performed by PLINK. When logistic regression analyses demonstrated patient-control differences in the allele frequencies of a specific haplotype, the phased haplotype was clustered to obtain the relative genotypes and carriers. The odd ratios (ORs) with corresponding 95% confident intervals (CIs) were used to quantify the associations between the clustered genotypes/carriers of the haplotypes and schizophrenia.

Based on the sample size, the QUANTO program showed that the study had an 80% power to detect an effect of OR ≥ 1.5 for a MAF ranging between 0.1 and 0.4 in the sample of cases-controls and OR ≥ 1.8 in the males and females sample, and assuming a priori a 1% risk for schizophrenia in the general population.

All the primary analyses were also supplemented by a series of secondary analyses that included gender, diagnostic subtypes, and age at onset. These secondary analyses were as follows: 1) Forward logistic regression analyses, with group (patients *vs.* controls) as the dependent variable and allele/haplotype (carriers *vs*. others), gender, and allele/haplotype by gender interaction as independent variables, were utilised to establish the presence of a putatively different effect of the allele/haplotype between the two sexes. Log-transformed age was also entered as covariate. The effects were expressed as ORs with their corresponding 95% CIs. 2). Multinomial logistic regression analyses, with illness status (control = -1, non-paranoid schizophrenia = 0, paranoid schizophrenia = 1) as the dependent variable and allele/haplotype presence, gender, and allele/haplotype by gender interaction as predictive variables and log-transformed age as covariate, were utilised to define the possible specific effects of the allele/haplotype in definite schizophrenia subtypes. The analyses were performed considering the controls as the reference category. 3) Factorial analyses of variance (ANOVA) of the log-transformed age of schizophrenia onset, also including the gender, were utilised to assess the allele/haplotype influence on the log-transformed onset of disorder and eventual interactions with the gender.

Goodness of fit was assessed by means of the Hosmer and Lemeshow test.

The gene-gene interactions between the most significant SNPs were assessed by logistic regression methods.

All of these analyses were performed using SPSS software, version 12.0 (SPSS Inc. Chicago, IL).

## Results

32 tagSNPs were analyzed in the statistical analyses.

### DAO

The six selected polymorphisms did not evidence patient-control differences in genotype, allele, or carrier frequencies (for details, see Additional file 1: Table S1). The same was true for the haplotype analysis.

There were 9 sliding windows for DAO gene. According to the LD panel (see Additional file 1: Table S2), we conducted the analyses considering the rs3741775 along with the other single SNPs, rs3918347 along with the other single SNPs and between them, setting a p value significant of 0.006 (0.05/9).

Secondary analyses of the haplotypes instead revealed effects related to gender and the diagnostic subtype of schizophrenia. In particular, haploview analyses showed a patient-control difference in the allele estimated GT diplotype (rs2070586 A/G-rs3741775 G/T) limited to females (p = 0.003, p_correct_ = 0.009; Table 1): after phased analysis and clusterisation, the genotype frequencies of the estimated/phased GT diplotype were different (p = 0.002), with the carriers more represented in female controls (p = 0.017, OR = 0.58, 95% CI: 0.37-0.90). No association was found in males (p = 0.92, OR = 0.98, 95% CI: 0.65-1.47). The male-female difference had been confirmed by the interaction between gender and the estimated/phased GT diplotype carrier (p = 0.003, OR = 0.43*,* 95% CI: 0.24-0.75).

**Table 1:** Estimated haplotype distributions for selected D-amino acid oxidase (DAO), protein phosphatise 3 catalytic subunit gamma isoform (PPP3CCC) and dystrobrevin-binding protein 1 (DTNBP1) single nucleotide polymorphisms in patients with schizophrenia and control subjects and in females and males samples

**DAO Haplotype (rs2070586|rs3741775)**							
	**Haplotype**	**Patients**^**a**^	**Controls**^**b**^	***χ***^**2**^	**DF**	**P**	**P correct**^**c**^
Total samples		N = 386	N = 476				
	GG	0.42	0.40	0.69	1	0.41	
	AT	0.21	0.19	1.40	1	0.24	
	GT	0.37	0.41	3.70	1	0.05	0.13
Females		N = 126	N = 270				
	GG	0.43	0.39	1.31	1	0.25	
	AT	0.23	0.17	3.88	1	0.05	0.11
	GT	0.33	0.44	8.65	1	0.003	0.009
Males		N = 260	N = 206				
	GG	0.41	0.41	0.01	1	0.92	
	AT	0.20	0.21	0.10	1	0.75	
	GT	0.39	0.38	0.03	1	0.86	
**PPP3CC Haplotype (rs4872499|rs11780915|rs13271367)**							
	**Haplotype**	**Patients**^**a**^	**Controls**^**b**^	***χ***^**2**^	**DF**	**P**	**P correct**^**c**^
Total samples		N = 383	N = 476				
	CGA	0.61	0.56	3.50	1	0.06	
	TAG	0.22	0.21	0.62	1	0.43	
	CAG	0.13	0.19	11.56	1	7x10^-4^	0.001
	others	0.03	0.03				
Females		N = 125	N = 269				
	CGA	0.60	0.57	0.63	1	0.43	
	TAG	0.22	0.17	2.65	1	0.10	
	CAG	0.12	0.20	7.60	1	0.006	0.04
	others	0.05	0.05				
Males		N = 258	N = 207				
	CGA	0.61	0.55	3.52	1	0.06	
	TAG	0.22	0.25	1.14	1	0.29	
	CAG	0.13	0.17	2.98	1	0.08	
	others	0.03	0.02				
**DTNBP1 Haplotype (rs6459409|rs9476886)**							
Total samples		N = 381	N = 473				
	CT	0.17	0.20	2.18	1	0.14	
	TT	0.13	0.12	0.78	1	0.38	
	TC	0.69	0.68	0.28	1	0.60	
Females		N = 125	N = 270				
	CT	0.20	0.17	0.62	1	0.43	
	TT	0.15	0.12	1.23	1	0.27	
	TC	0.65	0.70	1.84	1	0.17	
Males		N = 256	N = 203				
	CT	0.16	0.24	8.55	1	0.003	0.006
	TT	0.12	0.11	0.34	1	0.56	
	TC	0.71	0.65	3.77	1	0.05	0.14

^a^haplotype frequencies in schizophrenia patients.

^b^haplotype frequencies in control subjects.

DF = degree of freedom.

^c^Pcorrect after 10000 permutations.

Multinomial logistic regression analysis demonstrated the presence of an association between estimated/phased GT diplotype female carriers when paranoid schizophrenia patients were compared to healthy controls (p = 0.01*,* relative risk ratio = 0.52, 95% CI: 0.31-0.87, controls as reference category, Additional file 1: Table S3B). No significant results were observed when the non-paranoid patients were compared to the controls.

No significant effects emerged regardless of the genotype, or carrier stratified for diagnostic subtype and age at schizophrenia onset (consistently, p > .10).

### DAOA

The primary analyses of the 6 DAOA gene polymorphisms failed to detect any association (for details, see Additional file 1: Table S1); this was true also when the haplotype distributions were considered. All the secondary analyses were equally negative.

### PPP3CC

The eight polymorphisms chosen for the PPP3CC gene were unrelated to schizophrenia risk in genotype, allele, and carrier after multiple correction (for details see Additional file 1: Table S1).

There were 11 sliding windows for PPP3CC. According to LD panel (see Additional file 1: Table S2), we conducted the analyses considering rs4872499 along with two SNPs in the Block 1 and along with all SNPs of the Block 2, setting the p value to 0.005 (0.05/11).

In relation to the haploview analysis, a patient-control difference emerged in the allele frequency for the estimated CAG triplotype in the SNP window rs4872499 T/C-rs11780915 A/G-rs13271367 G/A (p = 7×10^-4^, p_correct_ = 0.001, Table 1). Similarly, the clustered genotype frequencies of the estimated/phased CAG triplotype differed between cases and controls (p = 0.004), with the carriers showing a higher frequency in the control population (p = 0.002, OR = 0.59, 95% CI: 0.43-0.82).

Secondary analyses supported a significant effect of single SNPs rs11780915 and rs13271367 in males group, with carrier A rs11780915 and carrier G rs13271367 more represented in patients group (p_correct_ = 0.02, OR = 1.79, 95% CI: 1.19-2.67 and p_correct_ = 0.04, OR = 1.70*,* 95% CI: 1.14-2.52 respectively).

Concerning the haplotype analysis, there was a significant effect of the estimated CAG triplotype in the female group, with p correct value of 0.04 for the allele frequencies (Table 1). According to the correction for sliding window approach, this difference was lost (p = 0.006). However significant results were also observed for the clustered genotype (p = 0.03). As for the carriers, overrepresentation was found in female controls (p = 0.01, OR = 0.53*,* 95% CI: 0.32-0.87) but not in males (p = 0.09, OR = 0.68*,* 95% CI: 0.43-1.06). This female-specific protective effect was corroborated to some degree by the interaction between the estimated/phased CAG triplotype carriers and gender (p = 0.02; OR = 0.52*;* 95% CI: 0.29-0.91).

Multinomial logistic regression analysis demonstrated the presence of an association between estimated/phased CAG triplotype female/total sample carriers when paranoid schizophrenia patients were compared to healthy controls (p = 0.04*,* relative risk ratio = 0.54, 95% CI: 0.30-0.96, controls as reference category, Additional file 1: Table S3B). No significant results were observed when the non-paranoid patients were compared to the controls.

The ANOVA analysis showed that age at the onset of schizophrenia was not accounted for by this haplotype (consistently, p > .10) or any of the single polymorphisms.

### DTNBP1

The single marker analysis of 12 tagSNPs in the total sample (for details, see Additional file 1: Table S1) failed to demonstrate patient-control differences in allele and genotype frequencies, both in the recessive and the dominant inheritance model. The haplotype analyses also did not support any association with schizophrenia.

When the SNP frequencies were analysed in relation to gender, protective effect in males was observed for rs6459409 (p_correct_ = 0.02, carrier C, OR = 0.55*,* 95% CI: 0.37-0.81).

Considering rs6459409 along with all other SNPs (see Additional file 1: Table S2), we calculated 11 sliding windows, setting a p value to 0.005 (0.05/11). The haploview analyses in males showed CC (rs10456773 C/T -rs6459409 C/T) and CT (rs6459409 C/T -rs9476886 T/C) allele haplotypes having some protective association with schizophrenia (p_correct_ = 0.03, p_correct_ = 0.006 respectively) (Table 1), whereas CT (rs10456773-rs6459409) haplotype showed a risk association (p_correct_ = 0.01).

Because the estimated/phased CT diplotype (rs6459409-rs9476886) was more significant compared to the others, the male carriers were also evaluated, and a protective effect on schizophrenia (p = 3×10^-4^*,* OR = 0.46*,* 95% CI: 0.30-0.70) emerged. No association was found in females (p = 0.44*,* OR = 1.20*,* 95% CI: 0.76-1.88). The presence of a significant interaction with gender (p = 5×10^-6^*,* OR = 3.69*,* 95% CI: 2.10-6.49) further supported these two findings.

The stratification of the polymorphisms (genotypes and carriers) and estimated/phased haplotypes (CT carriers vs. others) according to age of onset and diagnostic subtypes failed to detect significant associations (consistently, p > .10).

### Interaction effects

Due to the significant results for the estimated/phased *DAO* GT diplotype and *PPP3CC* CAG triplotype carriers in females, we verified whether the effects obtained were stronger through an interaction analysis. The results showed that the females carrying both the CAG and GT haplotypes had a 66% (p = 0.003, OR = 0.34, 95% CI: 0.17-0.70, Additional file 1: Table S3A) lower risk of developing schizophrenia relative to non-CAG or -GT carriers. All of the other interactions showed negative results.

## Discussion

The primary analyses did not demonstrate associations between schizophrenia and any of the 32 studied *DAOA*, *DAO*, *PPP3CC*, and *DTNBP1* SNPs. This finding is in line with other studies performed in larger samples [19-23].

The secondary analyses instead confirmed effects of the *DAO*, *PPP3CC*, and *DTNBP1* genes on schizophrenia susceptibility; these effects were mediated not only by the gender and paranoid/non-paranoid dichotomy but also by epistatic/interaction mechanisms.

The discrepancies between the primary and the secondary analyses may be reconciled assuming that the *DAO*, *PPP3CC*, and *DTNBP1* genes modulate the susceptibility for schizophrenia only under definite circumstances.

Gender-related differences in schizophrenia are far from a novel finding: an extensive literature indicates that males and females differ from each other in some psychopathological domains, treatment efficacy, and the presence of structural and brain abnormalities [24-26]. The influence of gender on schizophrenia has been related to oestrogens [25,26], neurodevelopment [27,28], and lateralization [29].

However, it is possible that in schizophrenia, as in many other complex disorders [30], several genes or genetic variations may affect susceptibility to the disorder in relation to the gender of the patient both for female or male specific protective or risk effects [13,31-33].

Present data concerning an influence of gender not only on the *DAO*, *DTNBP1*, and *PPP3CC* genes but also on the epistatic effect between the *DAO* and *PPP3CC* genes undoubtedly fit well within this scenario and have some specific antecedents.

In particular, in a Korean population, the *DAO* gene evidenced gender-specific differences in relation to the allele distributions and haplotypes of three SNPs, rs2070586, rs2070587, and rs3918347, which acted as risk or protective factors in relation to the sex of the patients [34]. The Korean study [34] and the current study differ in the haplotype combination involved; this discrepancy could be explained, as already reported [23], by ethnic background differences in the *DAO* gene, with variations between Asians and Caucasians in both allele frequencies and linkage patterns (http://hapmap.ncbi.nlm.nih.gov/index.html.en). Furthermore, animal studies have suggested that the *DAO* activity differs according to sex, most likely due to the effects of sex hormones [35,36]. Given the effect of oestradiol in reducing *DAO* activity in the liver tissues of female guinea pigs [35], oestrogens could have a protective effect on schizophrenia by preventing increases of *DAO* activity and subsequent NMDA hypofunction. In particular, the presence of a hormonally influenced transcriptional regulation of NMDA genes, mainly the *DAO* gene, could explain our results in females.

Since the significant interaction of gender and *DAO* and *PPP3CC* genes, an epistatic effect was performed and a 66% lower risk of developing schizophrenia was observed among females with the *PPP3CC* CAG triplotype and the *DAO* GT diplotype carriers as compared with non-CAG or -GT haplotype carriers of the same sex, whereas the CAG triplotype and the GT diplotype alone showed a 47% and a 42% lower risk for schizophrenia, respectively. Therefore, it could be hypothesised not only that the major protective effect of *DAO* could be mediated by the non-interaction with oestradiol [35,36], but also that the potentiation played by *PPP3CC* may be sustained by some interaction with the sex hormone pathway.

Other relevant findings come from the analysis of *DTNBP1* gene polymorphisms and haplotypes: the observation of an influence of *DTNBP1* on schizophrenia susceptibility in males could indicate the presence of sex-specific effect and/or of interactions with sex hormones.

With respect to the diagnostic subtype, the 42% and 47% excess of the estimated/phased *DAO* GT diplotype and *PPP3CC* CAG triplotype female carriers respectively found in controls compared to patients did not increase when exclusively female paranoid patients were considered (48% and 46% respectively). Therefore, the protective effect of the both *DAO* GT diplotype and *PPP3CC* CAG triplotype carriers is reasonably not paranoid-specific but comparable to schizophrenia as a whole. Similar results were obtained for *PPP3CC* CAG triplotype carriers and paranoid patients versus schizophrenia as whole (see Additional file 1: Table S3B). Although this putative involvement of *DAO* and *PPP3CC* in this specific subgroup was observed also for other genes related to the same glutamatergic/NMDAR regulation [37-40] associated with paranoid schizophrenia, a larger sample is needed to confirm this finding.

The present study requires some further comments. Although our SNPs are substantially informative due to their distribution covering the whole genes, they do not all correspond to those used in other studies. Thus, in the case of the analysis of the same SNP, our association can be attributed to stratification, even though we cannot exclude other confounding clinical phenotypes that manifest differently in male and female schizophrenia patients, such as treatment response, comorbid mood symptoms, and cognitive impairment, rather than a direct link between these genes and female/male schizophrenia. In the case of positive associations with different SNPs in the same gene, we cannot exclude the replica value because haplotype-specific functional variations have not been analysed. A recent GWAS study on bipolar patients showed that there is likely common genetic variation associated with the disorder near exons (±10 kb) that could be identified in larger studies and also provided a framework for assessing the potential for replication when combining results from multiple studies [41].

Moreover the major limitation of this paper is the relatively small sample size mainly after stratification for gender and diagnostic subtypes; however, because of we have based our study on the phenotype dissection strategy, we have computed the power for higher OR. This represents a suggestion for future replications in larger samples.

Finally, there is a formal possibility of type I error due to the limited number of samples available for analyses after different stratifications. However mainly in the case of stratifications by sex, significant results persist even after correction for multiple testing in the subgroups, and this makes it reasonable to assume that there could be only a low possibility of type I error in the results.

## Conclusions

This study represents the first evidence of association of 3 NMDA-receptor-mediated signalling genes, *DAO, DAOA, PPP3CC,* and *DTNBP1* in an Italian population. In particular *DAO*, *PPP3CC*, and *DTNBP1*, might be differentially involved in schizophrenia susceptibility according to gender and gene interaction mechanisms. Although the results are preliminary and needed replication in a larger sample, the present results underline the need for more systematic use of the phenotype “dissection” strategy and the search for interaction effects to strengthen the informative power of genetic association studies.

## Competing interests

The authors declare that they have no competing interests.

## Authors’ contributions

ES, CS conceived of the study, participated in its design and the coordination and acquisition of data, performed the statistical analyses, and co-wrote the manuscript; AM participated in the design of the study and enrolled and screened controls and patients and helped draft the manuscript; PV, AG, RP enrolled and screened patients; CB carried out all genetic analyses; PP performed the statistical analyses; MG conceived of the study, participated in its design and coordination, and helped draft the manuscript and critically reviewed it for intellectual content. All the authors read and approved the final manuscript.

## Pre-publication history

The pre-publication history for this paper can be accessed here:

http://www.biomedcentral.com/1471-2350/14/33/prepub

## Supplementary Material

Additional file 1: Table S1 Allele and genotype association analyses for single SNPs in D-amino acid oxidase, *DAO*, D-amino acid oxidase activator, *DAOA*, protein phosphatase 3 catalytic subunit gamma isoform, *PPP3CC* and dystrobrevin-binding protein 1, *DTNBP1* genes. **Table S2**. The linkage disequilibrium (LD) block structures of the D-amino acid oxidase, *DAO* (A); D-amino acid oxidase activator, *DAOA* (B); protein phosphatase 3 catalytic subunit gamma isoform, *PPP3CC* (C); and dystrobrevin-binding protein 1, *DTNBP1* (D) genes for the cases and controls. **Table S3**. Significant results from the secondary analyses for D-amino acid oxidase, *DAO*; protein phosphatase 3 catalytic subunit gamma isoform, *PPP3CC.* A. Interaction effect of *DAO* GT diplotype and *PPP3CC* CAG triplotype carriers. B. Multinomial logistic regression analyses in *DAO* GT diplotype and *PPP3CC* CAG triplotype carriers in paranoid/non paranoid sample. Click here for file
