# Supplementary material for: Schizophrenia susceptibility and NMDA-receptor mediated signalling: an association study involving 32 tagSNPs of DAO, DAOA, PPP3CC, and DTNBP1 genes
Source: BMC Med Genet. 2013 Mar 9;14:33. doi: 10.1186/1471-2350-14-33 (PMC3599832; doi:10.1186/1471-2350-14-33)
Supplement: Additional file 1: Table S1 — Allele and genotype association analyses for single SNPs in D-amino acid oxidase, DAO, D-amino acid oxidase activator, DAOA, protein phosphatase 3 catalytic subunit gamma isoform, PPP3CC and dystrobrevin-binding protein 1, DTNBP1 genes. Table S2. The linkage disequilibrium (LD) block structures of the D-amino acid oxidase, DAO (A); D-amino acid oxidase activator, DAOA (B); protein phosphatase 3 catalytic subunit gamma isoform, PPP3CC (C); and dystrobrevin-binding protein 1, DTNBP1 (D) genes for the cases and controls. Table S3. Significant results from the secondary analyses for D-amino acid oxidase, DAO; protein phosphatase 3 catalytic subunit gamma isoform, PPP3CC. A. Interaction effect of DAO GT diplotype and PPP3CC CAG triplotype carriers. B. Multinomial logistic regression analyses in DAO GT diplotype and PPP3CC CAG triplotype carriers in paranoid/non paranoid sample. [file 1471-2350-14-33-S1.doc]

**Quality control**

**After selection criteria, three (rs9558574 in DAOA, rs17733133 in PPP3CC, rs10456775 in DTNBP1) were excluded as it showed a significant deviation from HWE; whereas twelve (rs7967441 in DAO; rs2153674 in DAOA and; rs2469758, rs2461482 and rs17671456 in PPP3CC, and rs9370823, rs2619539, rs7768128, rs760761, rs2619521, rs13192791, rs7752070 in DTNBP1) were excluded for a completion rate <90%.**

**On a sample of about 200 subjects, we performed a replica of the 47 SNPs using the same technique SNPlex and the results indicated a Genotype Quality value of 97.5%. Moreover, after the comparison between the original and the replica plates, the 12 SNPs excluded for completation rate <90% in the total sample showed a call rate <80%.**

**Successively, on a subsample of approximately half of the whole sample, we tested the quality control by using another technique (Affymetrix Human Mapping GeneChip 6.0 arrays). We obtained a genotyping call rate of approximately >95%.**

**Table S1.** Allele and genotype association analyses for single SNPs in *D-amino acid oxidase, DAO,* *D-amino acid oxidase activator,* *DAOA*, *protein phosphatase 3 catalytic subunit gamma isoform,* *PPP3CC* and *dystrobrevin-binding protein 1*, *DTNBP1* genes.

| Genes | CHR | SNPs | Position (bp) | Function | A1/A2a | Test | Patients | Patients (N) | Controls | Controls (N) | χ^2^ | DF | UnadJb | OR | 95%CI |
| --- | --- | --- | --- | --- | --- | --- | --- | --- | --- | --- | --- | --- | --- | --- | --- |
| DAO | 12q24 | rs2070586 | 107801849 | Intron 1 | A/G | Genotypic | 22/120/240 | 382 | 17/143/311 | 471 | 2.54 | 2 | 0.28 |  |  |
|  |  |  |  |  |  | Allelic | 164/600 | 764 | 177/765 | 942 | 1.89 | 1 | 0.17 |  |  |
|  |  |  |  |  |  | DOM | 142/240 |  | 160/311 |  | 0.95 | 1 | 0.33 | 0.87 | 0.66-1.15 |
|  |  |  |  |  |  | REC | 22/360 |  | 17/454 |  | 2.23 | 1 | 0.14 | 1.63 | 0.85-3.12 |
|  |  | rs2070587 | 107801872 | Intron 1 | G/T | Genotypic | 23/130/219 | 372 | 24/152/273 | 449 | 0.45 | 2 | 0.80 |  |  |
|  |  |  |  |  |  | Allelic | 176/568 | 744 | 200/698 | 898 | 0.44 | 1 | 0.51 |  |  |
|  |  |  |  |  |  | DOM | 153/219 |  | 176/273 |  | 0.32 | 1 | 0.57 | 0.92 | 0.70-1.22 |
|  |  |  |  |  |  | REC | 23/349 |  | 24/425 |  | 0.26 | 1 | 0.61 | 1.17 | 0.65-2.10 |
|  |  | rs2111902 | 107802876 | Intron 1 | G/T | Genotypic | 42/183/152 | 377 | 63/219/180 | 462 | 1.19 | 2 | 0.55 |  |  |
|  |  |  |  |  |  | Allelic | 267/487 | 754 | 345/579 | 924 | 0.67 | 1 | 0.41 |  |  |
|  |  |  |  |  |  | DOM | 225/152 |  | 282/180 |  | 0.16 | 1 | 0.69 | 1.06 | 0.80-1.40 |
|  |  |  |  |  |  | REC | 42/335 |  | 63/399 |  | 1.18 | 1 | 0.28 | 0.79 | 0.52-1.20 |
|  |  | rs10861974 | 107806814 | Intron 3 | T/C | Genotypic | 21/137/222 | 380 | 29/192/252 | 473 | 2.26 | 2 | 0.32 |  |  |
|  |  |  |  |  |  | Allelic | 179/581 | 760 | 250/696 | 946 | 1.85 | 1 | 0.17 |  |  |
|  |  |  |  |  |  | DOM | 158/222 |  | 221/252 |  | 2.26 | 1 | 0.13 | 1.23 | 0.94-1.62 |
|  |  |  |  |  |  | REC | 21/359 |  | 29/444 |  | 0.14 | 1 | 0.71 | 0.90 | 0.50-1.60 |
|  |  | rs3741775 | 107807732 | Intron 4 | G/T | Genotypic | 65/194/124 | 383 | 74/203/160 | 437 | 1.80 | 2 | 0.41 |  |  |
|  |  |  |  |  |  | Allelic | 324/442 | 766 | 351/523 | 874 | 0.77 | 1 | 0.38 |  |  |
|  |  |  |  |  |  | DOM | 259/124 |  | 277/160 |  | 1.62 | 1 | 0.20 | 0.83 | 0.62-1.11 |
|  |  |  |  |  |  | REC | 65/318 |  | 74/363 |  | 0.00 | 1 | 0.99 | 1.00 | 0.70-1.14 |
|  |  | rs3918347 | 107817449 | Intron 10 | G/A | Genotypic | 41/173/167 | 381 | 57/212/201 | 470 | 0.40 | 2 | 0.82 |  |  |
|  |  |  |  |  |  | Allelic | 255/507 | 762 | 326/614 | 940 | 0.28 | 1 | 0.60 |  |  |
|  |  |  |  |  |  | DOM | 214/167 |  | 269/201 |  | 0.10 | 1 | 0.75 | 1.04 | 0.79-1.37 |
|  |  |  |  |  |  | REC | 41/340 |  | 57/413 |  | 0.39 | 1 | 0.53 | 0.87 | 0.57-1.34 |
| DAOA | 13q34 | rs1341402 | 104913510 | Upstream 5’UTR | C/T | Genotypic | 25/149/201 | 375 | 22/180/265 | 467 | 1.87 | 2 | 0.39 |  |  |
|  |  |  |  |  |  | Allelic | 199/551 | 750 | 224/710 | 934 | 1.44 | 1 | 0.23 |  |  |
|  |  |  |  |  |  | DOM | 174/201 |  | 202/265 |  | 0.83 | 1 | 0.36 | 0.87 | 0.66-1.14 |
|  |  |  |  |  |  | REC | 25/350 |  | 22/445 |  | 1.51 | 1 | 0.22 | 1.43 | 0.79-2.58 |
|  |  | rs1341403 | 104914808 | Upstream 5’UTR | A/C | Genotypic | 45/183/150 | 378 | 48/233/202 | 483 | 1.00 | 2 | 0.61 |  |  |
|  |  |  |  |  |  | Allelic | 273/483 | 756 | 329/637 | 966 | 0.79 | 1 | 0.38 |  |  |
|  |  |  |  |  |  | DOM | 228/150 |  | 281/202 |  | 0.40 | 1 | 0.53 | 0.92 | 0.70-1.22 |
|  |  |  |  |  |  | REC | 45/333 |  | 48/435 |  | 0.85 | 1 | 0.36 | 1.21 | 0.78-1.86 |
|  |  | rs6491961 | 104928380 | intron | C/T | Genotypic | 40/151/181 | 372 | 30/177/185 | 392 | 3.01 | 2 | 0.22 |  |  |
|  |  |  |  |  |  | Allelic | 231/513 | 744 | 237/547 | 784 | 0.12 | 1 | 0.73 |  |  |
|  |  |  |  |  |  | DOM | 332/40 |  | 362/30 |  | 2.20 | 1 | 0.14 | 1.45 | 0.88-2.39 |
|  |  |  |  |  |  | REC | 181/191 |  | 185/207 |  | 1.16 | 1 | 0.69 | 1.06 | 0.80-1.41 |
|  |  | rs9558571 | 104936367 | intron | T/C | Genotypic | 39/166/155 | 360 | 48/211/206 | 465 | 0.15 | 2 | 0.93 |  |  |
|  |  |  |  |  |  | Allelic | 244/476 | 720 | 307/623 | 930 | 0.14 | 1 | 0.71 |  |  |
|  |  |  |  |  |  | DOM | 205/155 |  | 259/206 |  | 0.13 | 1 | 0.72 | 0.95 | 0.72-1.26 |
|  |  |  |  |  |  | REC | 39/321 |  | 48/417 |  | 0.06 | 1 | 0.81 | 1.04 | 0.67-1.63 |
|  |  | rs778294 | 104940236 | intron | A/G | Genotypic | 32/144/202 | 378 | 27/207/245 | 479 | 4.02 | 2 | 0.13 |  |  |
|  |  |  |  |  |  | Allelic | 208/548 | 756 | 261/697 | 958 | 0.02 | 1 | 0.90 |  |  |
|  |  |  |  |  |  | DOM | 176/202 |  | 234/245 |  | 0.44 | 1 | 0.51 | 1.03 | 0.83-1.42 |
|  |  |  |  |  |  | REC | 32/346 |  | 27/452 |  | 2.64 | 1 | 0.10 | 1.58 | 0.93-2.68 |
|  |  | rs12864685 | 104941856 | 3' near gene | T/C | Genotypic | 23/120/225 | 368 | 16/155/307 | 478 | 4.12 | 2 | 0.13 |  |  |
|  |  |  |  |  |  | Allelic | 166/570 | 736 | 187/769 | 956 | 2.26 | 1 | 0.13 |  |  |
|  |  |  |  |  |  | DOM | 143/225 |  | 171/307 |  | 0.85 | 1 | 0.36 | 0.86 | 0.65-1.14 |
|  |  |  |  |  |  | REC | 23/345 |  | 16/462 |  | 3.98 | 1 | 0.05* | 1.90 | 0.99-3.66 |
| PPP3CC | 8p21.3 | rs4872499 | 22349251 | 5’ near gene | T/C | Genotypic | 24/132/213 | 369 | 21/170/280 | 471 | 1.73 | 2 | 0.42 |  |  |
|  |  |  |  |  |  | Allelic | 180/558 | 738 | 212/730 | 942 | 0.82 | 1 | 0.36 |  |  |
|  |  |  |  |  |  | DOM | 156/213 |  | 191/280 |  | 0.25 | 1 | 0.61 | 0.93 | 0.71-1.23 |
|  |  |  |  |  |  | REC | 24/345 |  | 21/450 |  | 1.71 | 1 | 0.19 | 1.49 | 0.82-2.72 |
|  |  | rs11780915 | 22360962 | intron 1 | A/G | Genotypic | 51/146/152 | 349 | 75/201/160 | 436 | 3.90 | 2 | 0.14 |  |  |
|  |  |  |  |  |  | Allelic | 248/450 | 698 | 351/521 | 872 | 3.66 | 1 | 0.06 |  |  |
|  |  |  |  |  |  | DOM | 197/152 |  | 276/160 |  | 3.80 | 1 | 0.05* | 1.33 | 1.00-1.77 |
|  |  |  |  |  |  | REC | 51/298 |  | 75/361 |  | 0.96 | 1 | 0.33 | 0.82 | 0.56-1.21 |
|  |  | rs13271367 | 22380522 | intron 1 | G/A | Genotypic | 53/171/150 | 374 | 86/223/154 | 463 | 5.35 | 2 | 0.07 |  |  |
|  |  |  |  |  |  | Allelic | 277/471 | 748 | 395/531 | 926 | 5.45 | 1 | 0.02* |  |  |
|  |  |  |  |  |  | DOM | 224/150 |  | 309/154 |  | 4.19 | 1 | 0.04* | 1.34 | 1.01-1.80 |
|  |  |  |  |  |  | REC | 53/321 |  | 86/377 |  | 2.90 | 1 | 0.09 | 0.72 | 0.50-1.05 |
|  |  | rs7827118 | 22390614 | intron 3 | G/T | Genotypic | 75/182/107 | 364 | 117/223/125 | 465 | 2.47 | 2 | 0.29 |  |  |
|  |  |  |  |  |  | Allelic | 332/396 | 728 | 457/473 | 930 | 2.05 | 1 | 0.15 |  |  |
|  |  |  |  |  |  | DOM | 257/107 |  | 340/125 |  | 0.64 | 1 | 0.42 | 1.13 | 0.83-1.53 |
|  |  |  |  |  |  | REC | 75/289 |  | 117/348 |  | 2.38 | 1 | 0.12 | 0.77 | 0.55-1.07 |
|  |  | rs2469749 | 22413543 | intron 4 | T/C | Genotypic | 48/177/155 | 380 | 49/185/222 | 456 | 5.23 | 2 | 0.07 |  |  |
|  |  |  |  |  |  | Allelic | 273/487 | 760 | 283/629 | 912 | 4.47 | 1 | 0.03* |  |  |
|  |  |  |  |  |  | DOM | 225/155 |  | 234/222 |  | 5.22 | 1 | 0.02* | 0.73 | 0.55-0.96 |
|  |  |  |  |  |  | REC | 48/332 |  | 49/407 |  | 0.72 | 1 | 0.40 | 1.20 | 0.79-1.83 |
|  |  | rs2469755 | 22416157 | intron 4 | A/G | Genotypic | 77/177/123 | 377 | 107/230/138 | 475 | 1.40 | 2 | 0.50 |  |  |
|  |  |  |  |  |  | Allelic | 331/423 | 754 | 444/506 | 950 | 1.37 | 1 | 0.24 |  |  |
|  |  |  |  |  |  | DOM | 254/123 |  | 337/138 |  | 1.26 | 1 | 0.26 | 1.17 | 0.88-1.57 |
|  |  |  |  |  |  | REC | 77/300 |  | 107/368 |  | 0.55 | 1 | 0.46 | 0.87 | 0.62-1.21 |
|  |  | rs2461483 | 22442424 | Intron 9 | T/C | Genotypic | 77/174/119 | 370 | 103/216/129 | 448 | 1.26 | 2 | 0.53 |  |  |
|  |  |  |  |  |  | Allelic | 328/412 | 740 | 422/474 | 896 | 1.26 | 1 | 0.26 |  |  |
|  |  |  |  |  |  | DOM | 251/119 |  | 319/129 |  | 1.09 | 1 | 0.30 | 1.17 | 0.87-1.58 |
|  |  |  |  |  |  | REC | 77/293 |  | 103/345 |  | 0.56 | 1 | 0.45 | 0.88 | 0.63-1.23 |
|  |  | rs2469776 | 22459396 | 3’near gene | C/T | Genotypic | 76/181/120 | 377 | 112/222/137 | 471 | 1.79 | 2 | 0.41 |  |  |
|  |  |  |  |  |  | Allelic | 333/421 | 754 | 446/496 | 942 | 1.71 | 1 | 0.19 |  |  |
|  |  |  |  |  |  | DOM | 257/120 |  | 334/137 |  | 0.75 | 1 | 0.39 | 1.14 | 0.85-1.53 |
|  |  |  |  |  |  | REC | 76/301 |  | 112/359 |  | 1.59 | 1 | 0.21 | 0.81 | 0.58-1.12 |
| DTNBP1 | 6p22.3 | rs909626 | 15519914 | near 3'UTR | A/G | Genotypic | 21/108/212 | 341 | 19/152/285 | 456 | 1.71 | 2 | 0.43 |  |  |
|  |  |  |  |  |  | Allelic | 150/532 | 682 | 190/722 | 912 | 0.31 | 1 | 0.58 |  |  |
|  |  |  |  |  |  | DOM | 129/212 |  | 171/285 |  | 0.01 | 1 | 0.92 | 0.99 | 0.74-1.32 |
|  |  |  |  |  |  | REC | 21/320 |  | 19/437 |  | 1.62 | 1 | 0.20 | 1.51 | 0.80-2.85 |
|  |  | rs13198533 | 15517684 | near 3'UTR | T/C | Genotypic | 20/130/229 | 379 | 27/187/257 | 471 | 2.98 | 2 | 0.23 |  |  |
|  |  |  |  |  |  | Allelic | 170/588 | 758 | 241/701 | 942 | 2.28 | 1 | 0.13 |  |  |
|  |  |  |  |  |  | DOM | 150/229 |  | 214/257 |  | 2.94 | 1 | 0.09 | 1.27 | 0.96-1.66 |
|  |  |  |  |  |  | REC | 20/359 |  | 27/444 |  | 0.08 | 1 | 0.77 | 0.91 | 0.50-1.66 |
|  |  | rs1047631 | 15523101 | 3'UTR | G/A | Genotypic | 7/88/251 | 346 | 5/99/340 | 444 | 2.26 | 2 | 0.32 |  |  |
|  |  |  |  |  |  | Allelic | 102/590 | 692 | 109/779 | 888 | 2.04 | 1 | 0.15 |  |  |
|  |  |  |  |  |  | DOM | 95/251 |  | 104/340 |  | 1.68 | 1 | 0.20 | 0.81 | 0.58-1.12 |
|  |  |  |  |  |  | REC | 7/339 |  | 5/439 |  | 1.05 | 1 | 0.31 | 1.81 | 0.57-5.76 |
|  |  | rs742106 | 15524480 | intron 9 | T/C | Genotypic | 45/159/148 | 352 | 63/192/167 | 422 | 0.93 | 2 | 0.63 |  |  |
|  |  |  |  |  |  | Allelic | 249/455 | 704 | 318/526 | 844 | 0.88 | 1 | 0.35 |  |  |
|  |  |  |  |  |  | DOM | 204/148 |  | 255/167 |  | 0.49 | 1 | 0.49 | 1.11 | 0.83-1.47 |
|  |  |  |  |  |  | REC | 45/307 |  | 63/359 |  | 0.74 | 1 | 0.39 | 0.83 | 0.55-1.26 |
|  |  | rs16876575 | 15525157 | intron 8 | T/C | Genotypic | 16/104/250 | 370 | 13/154/292 | 459 | 3.74 | 2 | 0.15 |  |  |
|  |  |  |  |  |  | Allelic | 136/604 | 740 | 180/738 | 918 | 0.40 | 1 | 0.53 |  |  |
|  |  |  |  |  |  | DOM | 120/250 |  | 167/292 |  | 1.41 | 1 | 0.23 | 1.19 | 0.89-1.59 |
|  |  |  |  |  |  | REC | 16/354 |  | 13/446 |  | 1.35 | 1 | 0.25 | 1.55 | 0.74-3.27 |
|  |  | rs4712253 | 15526417 | intron 8 | T/C | Genotypic | 70/174/128 | 372 | 81/236/156 | 473 | 0.88 | 2 | 0.64 |  |  |
|  |  |  |  |  |  | Allelic | 314/430 | 744 | 398/548 | 946 | 0.00 | 1 | 0.96 |  |  |
|  |  |  |  |  |  | DOM | 244/128 |  | 317/156 |  | 0.19 | 1 | 0.66 | 1.06 | 0.80-1.41 |
|  |  |  |  |  |  | REC | 70/302 |  | 81/392 |  | 0.52 | 1 | 0.47 | 1.14 | 0.80-1.62 |
|  |  | rs4236167 | 15533951 | intron 7 | T/C | Genotypic | 59/204/111 | 374 | 69/237/157 | 463 | 1.70 | 2 | 0.43 |  |  |
|  |  |  |  |  |  | Allelic | 322/426 | 748 | 375/551 | 926 | 1.11 | 1 | 0.29 |  |  |
|  |  |  |  |  |  | DOM | 263/111 |  | 306/157 |  | 1.70 | 1 | 0.19 | 0.82 | 0.61-1.10 |
|  |  |  |  |  |  | REC | 59/315 |  | 69/394 |  | 0.12 | 1 | 0.73 | 1.07 | 0.73-1.56 |
|  |  | rs10456773 | 15537325 | intron 7 | C/T | Genotypic | 22/109/249 | 380 | 17/160/293 | 470 | 4.40 | 2 | 0.11 |  |  |
|  |  |  |  |  |  | Allelic | 153/607 | 760 | 194/746 | 940 | 0.07 | 1 | 0.80 |  |  |
|  |  |  |  |  |  | DOM | 131/249 |  | 177/293 |  | 0.92 | 1 | 0.34 | 1.15 | 0.87-1.53 |
|  |  |  |  |  |  | REC | 22/358 |  | 17/453 |  | 2.27 | 1 | 0.13 | 1.63 | 0.85-3.12 |
|  |  | rs9296983 | 15555426 | intron 7 | A/G | Genotypic | 22/143/211 | 376 | 27/185/258 | 470 | 0.16 | 2 | 0.93 |  |  |
|  |  |  |  |  |  | Allelic | 187/565 | 752 | 239/701 | 940 | 0.07 | 1 | 0.79 |  |  |
|  |  |  |  |  |  | DOM | 165/211 |  | 212/258 |  | 0.13 | 1 | 0.72 | 1.05 | 0.80-1.38 |
|  |  |  |  |  |  | REC | 22/354 |  | 27/443 |  | 0.00 | 1 | 0.95 | 1.02 | 0.57-1.82 |
|  |  | rs9358063 | 15565031 | intron 7 | C/T | Genotypic | 73/189/99 | 361 | 93/244/128 | 465 | 0.01 | 2 | 1.00 |  |  |
|  |  |  |  |  |  | Allelic | 335/387 | 722 | 430/500 | 930 | 0.00 | 1 | 0.95 |  |  |
|  |  |  |  |  |  | DOM | 262/99 |  | 337/128 |  | 0.00 | 1 | 0.97 | 0.99 | 0.73-1.35 |
|  |  |  |  |  |  | REC | 73/288 |  | 93/372 |  | 0.00 | 1 | 0.99 | 1.00 | 0.71-1.41 |
|  |  | rs6459409 | 15636853 | intron 4 | C/T | Genotypic | 14/103/253 | 370 | 15/161/289 | 465 | 4.42 | 2 | 0.11 |  |  |
|  |  |  |  |  |  | Allelic | 131/609 | 740 | 191/739 | 930 | 2.13 | 1 | 0.14 |  |  |
|  |  |  |  |  |  | DOM | 117/253 |  | 176/289 |  | 3.51 | 1 | 0.06 | 1.33 | 0.99-1.77 |
|  |  |  |  |  |  | REC | 14/356 |  | 15/450 |  | 0.19 | 1 | 0.66 | 1.17 | 0.56-2.46 |
|  |  | rs9476886 | 15661461 | intron 1 | T/C | Genotypic | 40/148/185 | 373 | 47/203/216 | 466 | 1.29 | 2 | 0.53 |  |  |
|  |  |  |  |  |  | Allelic | 228/518 | 746 | 297/635 | 932 | 0.33 | 1 | 0.57 |  |  |
|  |  |  |  |  |  | DOM | 188/185 |  | 250/216 |  | 0.87 | 1 | 0.35 | 1.14 | 0.87-1.50 |
|  |  |  |  |  |  | REC | 40/333 |  | 47/419 |  | 0.09 | 1 | 0.76 | 1.06 | 0.68-1.66 |

^a^ A1 = minor allele

^b^UnadJ = uncorrected p-value

The values * were not significant after 10000 permutation/Bonferroni correction

**Table S2.** The linkage disequilibrium (LD) block structures of the D-amino acid oxidase, *DAO* (A); D-amino acid oxidase activator, *DAOA* (B); protein phosphatase 3 catalytic subunit gamma isoform, *PPP3CC* (C)*;*  and dystrobrevin-binding protein 1, *DTNBP1* (D) genes for the cases and controls.

**A B**

**
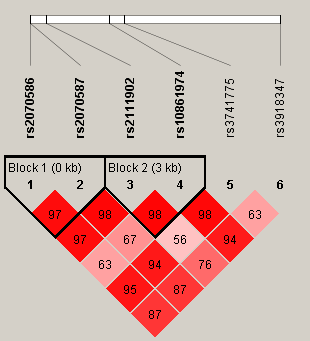

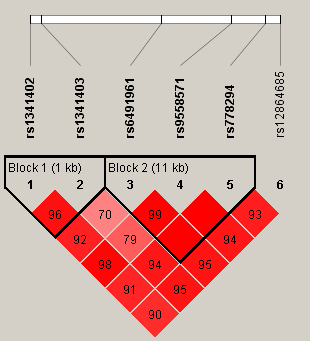
**

**C D**

**
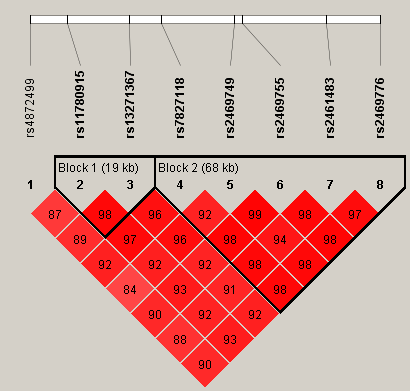

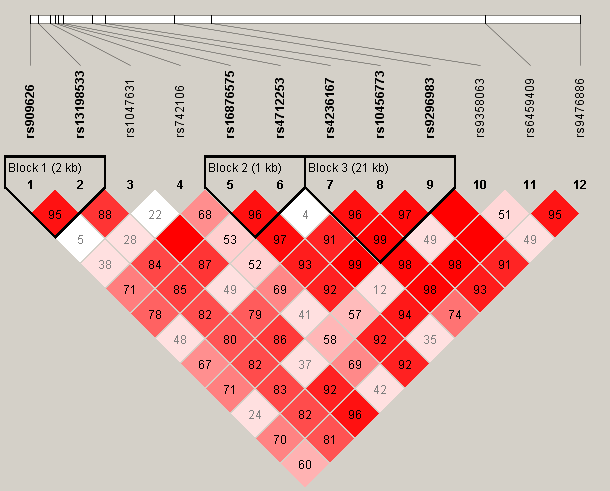
**

The LD measures are indicated as D’. The shades of red indicate the magnitudes of the D’ values, and red is equal to 100%.

**Table S3.** Significant results from the secondary analyses for D-amino acid oxidase, *DAO*; protein phosphatase 3 catalytic subunit gamma isoform, *PPP3CC*

A. Interaction effect of *DAO* GT diplotype and *PPP3CC* CAG triplotype carriers.

|  | **Total** |  |  |  |  | **Females** |  |  |  |  | **Males** |  |  |  |  |
| --- | --- | --- | --- | --- | --- | --- | --- | --- | --- | --- | --- | --- | --- | --- | --- |
|  | **Patients** | **Controls** | **p** | **OR** | **95%CI** | **Patients** | **Controls** | **p** | **OR** | **95%CI** | **Patients** | **Controls** | **p** | **OR** | **95%CI** |
| DAO GT carriers | 213 | 284 | 0.1 | 0.78 | 0.58-1.04 | 70 | 167 | 0.018 | 0.58 | 0.37-0.90 | 143 | 117 | 0.92 | 0.98 | 0.65-1.47 |
| others | 144 | 149 |  |  |  | 56 | 79 |  |  |  | 88 | 70 |  |  |  |
|  | 357 | 433 |  |  |  | 126 | 246 |  |  |  | 231 | 187 |  |  |  |
| PPP3CC CAG carriers | 77 | 152 | 0.002 | 0.59 | 0.43-0.82 | 29 | 91 | 0.01 | 0.53 | 0.32-0.87 | 48 | 61 | 0.09 | 0.68 | 0.43-1.06 |
| others | 273 | 312 |  |  |  | 95 | 170 |  |  |  | 178 | 142 |  |  |  |
|  | 350 | 464 |  |  |  | 124 | 261 |  |  |  | 226 | 203 |  |  |  |
| CAG+GT carriers | 45 | 83 | 0.003 | 0.50 | 0.31-0.79 | 16 | 50 | 0.003 | 0.34 | 0.17-0.70 | 29 | 33 | 0.54 | 0.81 | 0.42-1.56 |
| others | 107 | 96 |  |  |  | 42 | 46 |  |  |  | 65 | 50 |  |  |  |
|  | 152 | 179 |  |  |  | 58 | 96 |  |  |  | 94 | 83 |  |  |  |

The analyses were corrected according to log age and, in the total sample, according to sex

B. Multinomial logistic regression analyses in *DAO* GT diplotype and *PPP3CC* CAG triplotype carriers in paranoid/non paranoid sample.

|  |  | **Total** |  |  |  | **Females** |  |  |  | **Males** |  |  |  |
| --- | --- | --- | --- | --- | --- | --- | --- | --- | --- | --- | --- | --- | --- |
|  |  | **N** | **p** | **RRR** | **95%CI** | **N** | **p** | **RRR** | **95%CI** | **N** | **p** | **RRR** | **95%CI** |
| DAO GT carriers |  |  |  |  |  |  |  |  |  |  |  |  |  |
|  | paranoid | 221 | 0.04 | 0.70 | 0.50-0.99 | 80 | 0.01 | 0.52 | 0.31-0.87 | 141 | 0.74 | 1.08 | 0.68-1.71 |
|  | non paranoid | 136 | 0.76 | 1.06 | 0.71-1.60 | 46 | 0.31 | 1.40 | 0.73-2.71 | 90 | 0.51 | 0.83 | 0.49-1.42 |
| PPP3CC CAG carriers |  |  |  |  |  |  |  |  |  |  |  |  |  |
|  | paranoid | 219 | 0.04 | 0.54 | 0.30-0.96 | 79 | 0.04 | 0.54 | 0.30-0.96 | 140 | 0.11 | 1.52 | 0.90-2.55 |
|  | non paranoid | 132 | 0.09 | 1.92 | 0.91-4.05 | 45 | 0.09 | 1.92 | 0.90-4.07 | 87 | 0.30 | 1.41 | 0.77-2.58 |

The analyses were corrected according to log age and, in the total sample, according to sex

The controls sample was used as reference category:

N=433 for the total sample; N=246 for the females group; N=187 for the males group for *DAO*

N=465 for the total sample; N=263 for the females group; N=202 for the males group for *PPP3CC*
